# Sensitive Parameters of Dynamic Excitation on Fuze Airflow-Induced Acoustic Generator

**DOI:** 10.3390/mi12091033

**Published:** 2021-08-28

**Authors:** Zhipeng Li, Jinghao Li, Hejuan Chen

**Affiliations:** School of Mechanical Engineering, Nanjing University of Science and Technology, Nanjing 210094, China; Leejh1997@njust.edu.cn

**Keywords:** airflow-induced acoustic generator, fluid dynamic sound source, orthogonal test, sound pressure frequency

## Abstract

This paper aims at the power generation requirements of the fuze airflow-induced acoustic generator, analyzes the influence of structural parameters on the fluid power sound source, which is related to the power generation performance and use performance of the generator. In this paper, the orthogonal experiment method is used to study the sensitive parameters that control fluid dynamic sound sources. The results show that the annulus, the confronting distance, and cavity length can all have an impact on the sound pressure amplitude, and the sound pressure amplitude is most sensitive to the change of the confronting distance. However, the length of the resonant cavity has the most significant effect on the sound pressure frequency. The size of the annulus has a weak effect on the sound pressure frequency, and the confronting distance has almost no effect on the sound pressure frequency. The optimal combination scheme with the highest output power is selected according to the sensitive parameters. In addition, the empirical formula for the vibration frequency of the airflow-induced acoustic generator in the short resonant cavity was revised, and the influence of the annular gap on the vibration frequency was added, and the influence factor *α* = 0.3 was determined. The corrected frequency empirical formula has the smallest error between the theoretical value and the experimental value, and can be used as an effective method for estimating the vibration frequency. This provides a reference for the engineering design of the fuze airflow-induced acoustic generator, which has high military value and application prospects.

## 1. Introduction

From the point of view of acoustics, a source is a region of space, in contact with the fluid medium where new acoustic energy is being generated, to be radiated outward as sound waves [1,2]. As a kind of clean and renewable energy, acoustic energy is widely used in the environment, and the research of the acoustic energy harvester has also attracted the attention of scholars [3]. 

The acoustic energy harvester is usually composed of a resonant cavity, a diaphragm, and a transducing material [4]. The fluid dynamic sound source is a necessary condition for the fuze airflow-induced acoustic generator to generate electricity. It is affected by the geometry of the resonant cavity and the incoming flow parameters [5]. The current mainstream resonators include Helmholtz resonators and quarter-wave resonators [6,7]. Helmholtz resonant cavity is the most widely used cavity structure. In order to obtain greater sound pressure amplification, the improvement of Helmholtz resonant cavity has become a research hotspot [8,9,10,11,12,13,14]. The quarter-wavelength resonant cavity is conducive to the miniaturization of the structure. Optimizing the geometry of the resonant cavity or designing the coupling model can effectively increase the sound pressure amplification factor [15,16]. For example, Izhar et al. designed a three-degrees-of-freedom acoustic energy harvester using the Helmholtz resonator [17]. The purpose of increasing the degrees of freedom is to expand the narrow frequency bandwidth and improve the energy harvesting efficiency of the acoustic energy harvester. Starting from the cavity structure design, Khan et al. changed the structure of the traditional circular Helmholtz resonant cavity and designed a Helmholtz resonant cavity with a cone structure [17,18]. Through the test, the tapered Helmholtz resonator can better amplify the sound pressure under the same conditions, the voltage obtained is increased by 33.33%, and the output power is increased by 76.26%. Pillai et al. designed a Helmholtz resonator with a tapered neck structure [19], and experimentally verified that the tapered neck can increase the sound pressure magnification and help the sound energy harvester output a higher voltage. 

The acoustic energy harvester based on the Helmholtz resonant cavity structure requires a large-volume cavity, so the volume of the harvester is relatively large. Therefore, due to the miniaturization design requirements of the structure, the design of the acoustic energy harvester based on the quarter-wavelength resonator has also received the attention of scholars from all over the world. For example, Yuan et al. studied the spiral structure from the perspective of cochlear bionics and designed a new type of acoustic energy collector with spiral structure [20]. The design of the spiral tube is similar to the principle of the quarter-wavelength tube, but the use of the spiral structure makes the structure smaller, and the resonance frequency is not much different from that of the quarter-wavelength tube, so it is more suitable for small volume, low-frequency sound energy collection. Zou et al. designed a jet acoustic energy collector based on a quarter-wavelength tube [21]. The open end of the quarter-wavelength tube is designed as a wedge structure. The wedge structure can better cause the instability of the jet and produce a larger sound pressure amplitude. Li et al. further studied the conditions of edge sound generation [22], established a vibration model of the fuze airflow-induced acoustic generator, and explained the influence of edge sound on power generation performance through experiments. Zhou et al. designed a flexible tube acoustic energy collector [23], directly using piezoelectric materials to design a quarter wavelength resonator, which makes the flexible piezoelectric tube vibrate and generate electricity according to the radial radiation of sound pressure. Through the above literature, it is found that the resonator geometry and incoming flow parameters are the keys to the acoustic energy collection. The quarter-wavelength resonant cavity is more suitable for the acoustic energy collection device with a small volume structure. However, the analysis of the sensitive parameters of sound wave generation is mostly for single factor analysis, and few analyses of the influence of multiple factors on the hydrodynamic sound source. 

The fuze airflow-induced acoustic generator is a kind of sound energy harvester. At the same time, it is also a physical fuze power source, which uses high-speed jets to generate hydrodynamic sound sources, and then converts sound energy into electrical energy through the positive piezoelectric effect. The fuze airflow-induced acoustic generator works in external ballistics, with small structural size and high output power, which can effectively solve the contradiction between the small size and high power output of modern fuze power supplies. Therefore, if the output power can be increased by improving the driving performance of the airflow-induced acoustic piezoelectric generator and supplied to modern fuzes, it will have high military value and application prospects.

The structural parameters and the incoming flow velocity of the fuze airflow-induced acoustic generator are the keys to the generation of the fluid dynamic sound source and the magnitude of the exciting force, which directly control the output performance of the generator. In order to produce a stable hydrodynamic sound source of greater magnitude and increase the output power of the generator, it is necessary to study the sensitive parameters that control the generation of sound waves. This paper focuses on the process of airflow induced acoustic excitation which provides a theoretical basis for the engineering design of an airflow induced acoustic generator. Through the orthogonal experimental method, we analyzed the influence law of multiple structural parameters of air flow induced acoustic generator on hydrodynamic sound source, determined its sensitive parameters, and finally put forward the optimization scheme of frequency formula.

This paper first introduces the working principle of the fuze airflow-induced acoustic generator, then analyzes the structural parameters that affect the generation of the hydrodynamic sound source, obtains its sensitive parameters through orthogonal experiments, and finally proposes an empirical formula optimization scheme.

## 2. The Working Principle of Fuze Airflow-Induced Acoustic Generator

**Figure 1:**
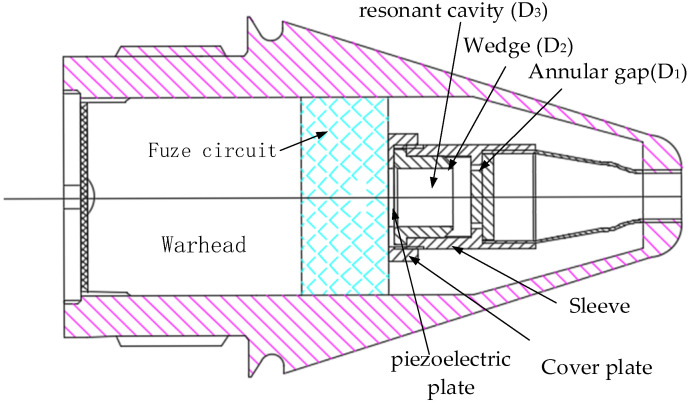
Experimental assembly drawing.

The airflow-induced acoustic generator consists of only five parts: sleeve, annular gap, resonant cavity, piezoelectric plate and cover plate. It is generally installed on the head of the projectile, and the airflow enters the excitation mechanism of the generator through the air inlet, forming a fluid power sound source and then exciting the piezoelectric transducer mechanism to generate electricity. The experimental assembly diagram is shown in Figure 1.

**Figure 2:**
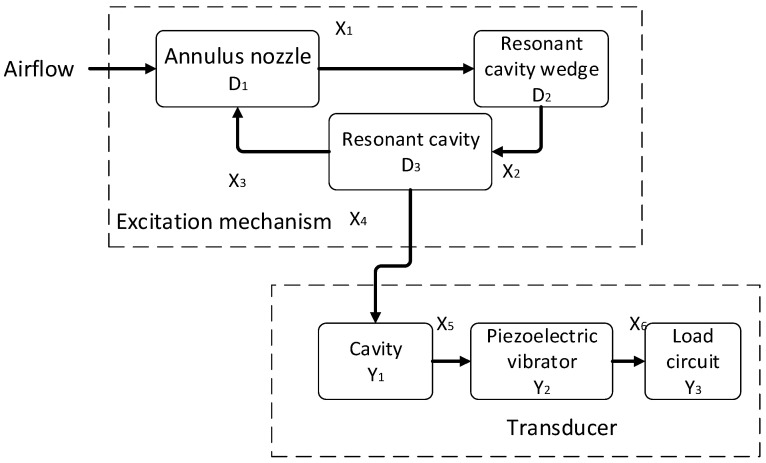
Working principle diagram of airflow induced acoustic generator.

The fuze airflow-induced acoustic generator can be divided into two mechanisms, namely the excitation mechanism and the energy conversion mechanism, as shown in Figure 2. The excitation mechanism includes annulus nozzle (*D*_1_), resonant cavity wedge (*D*_2_) and resonant cavity (*D*_3_). The energy conversion mechanism includes a cavity (Y_1_), a piezoelectric vibrator (*Y*_2_) and a conditioning circuit (*Y*_3_). $X_{1}-X_{6}$ represent the six stages of operation of the fuze airflow-induced acoustic generator, where X_1_ represents vortex generation, *X*_2_ represents acoustic excitation, *X*_3_ represents acoustic feedback, *X_4_* represents acoustic propagation, *X*_5_ represents acoustic radiation, and *X*_6_ represents energy conversion. The working process of the system can be described as: Vortex shedding phenomenon (*X*_1_) caused by external air flow through *D*_1_. After the shedding vortex collides with *D*_2_, sound waves are excited (*X*_2_). When the vibration frequency of the acoustic signal is inconsistent with the acoustic modal frequency of the resonant cavity (*D*_3_), the acoustic signal will be fed back to *D_1_*, thus affecting the vortex shedding frequency, which makes the shedding frequency consistent with the acoustic modal frequency of the cavity. When the vibration frequency of the acoustic signal is consistent with the acoustic modal frequency of the resonant cavity (*D*_3_), the acoustic resonance is formed, and the acoustic wave amplitude increases and propagates in *Y*_1_. The sound wave excites radiation in the cavity (*X*_5_) to excite *Y*_2_ vibration. *Y*_2_ vibration produces a positive piezoelectric effect, which converts the vibration excitation into electrical energy to supply *Y*_3_. 

According to the research on the power generation characteristics of the airflow-induced acoustic generator, the generator output power can be expressed as [24]:

(1) $$P_{e}=\frac{m\varepsilon_{e}X^{2}\omega_{n}{}^{3}\left(1+4\varepsilon^{2}\right)}{4\varepsilon^{2}}$$

where *m* represents the equivalent mass of the piezoelectric vibrator, *X* represents the sound pressure vibration amplitude, $\omega_{n}$ represents the sound pressure vibration angular frequency, ε represents damping, $\varepsilon_{p}$ represents mechanical damping, $\varepsilon_{e}$ represents electromechanical damping, and $\varepsilon =\varepsilon_{p}+\varepsilon_{e}$.

When the piezoelectric vibrator obtains the maximum electric power, the maximum power can be expressed as [24]:

(2) $$P_{e}=\frac{mX^{2}\omega_{n}{}^{3}\left(1+16\varepsilon_{e}{}^{2}\right)}{16\varepsilon_{e}}$$

Assuming that the strength of the piezoelectric vibrator is sufficient, and the mass and electromechanical damping are certain, the following conclusion can be drawn from Equation (2):

1. When the sound pressure frequency is constant, the output power of the fuze airflow-induced acoustic generator increases linearly with the square of the sound pressure amplitude.
2. When the sound pressure amplitude is constant, the output power of the fuze airflow-induced acoustic generator increases linearly with the third power of the sound pressure frequency.

Therefore, studying the influence of the sensitive parameters of the excitation mechanism of the fuze airflow-induced acoustic generator on the acoustic wave performance is very important to the design of the fuze airflow-induced acoustic generator.

## 3. Analysis of Sensitive Parameters of Fuze Airflow-Induced Acoustic Generator

**Figure 3:**
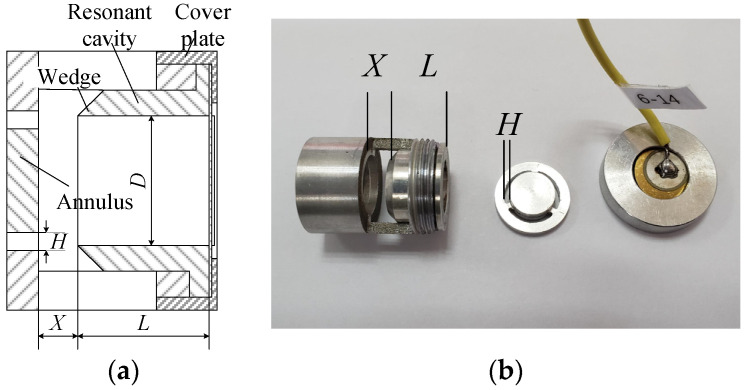
Structural parameters of airflow induced acoustic generator. (**a**) Structure diagram, (**b**) Physical exploded diagram.

According to the structural analysis of the experimental prototype in Figure 3, the airflow-induced acoustic generator mainly includes the following four parameters, the confronting distance *X* from the annulus to the resonant cavity, the length *L* of the resonant cavity, the distance *H* of the annulus gap size, and the diameter *D* of the resonant cavity.

For small spacing and short cavity structure, the calculation of acoustic modal frequency is based on the pulsating edge sound propagating in the cavity without considering the heat conversion in the tube. The sound wave propagates in the cavity, and the disturbance flow field deviates from its average value, which can be expressed as: $p=\bar{p}+\overset{\prime }{p}$, $\rho =\bar{\rho }+\overset{\prime }{\rho }$, where *p* represents pressure *ρ* represents density, $\bar{p}$ represents Reynolds time-average pressure, $\bar{\rho }$ represents Reynolds time-average density, $\overset{\prime }{p}$ represents pressure oscillation component, $\overset{\prime }{\rho }$ represents density oscillation component. The three-dimensional acoustic wave equation can be expressed as [25]:

(3) $$\frac{1}{c^{2}}\frac{\partial^{2}\overset{\prime }{p}}{\partial t^{2}}-\nabla^{2}\overset{\prime }{p}=0$$

Converting the three-dimensional acoustic wave equation to the one-dimensional standing wave equation can be obtained from Formula (4).

(4) $$\{\begin{array}{l} \overset{\prime }{p}=Ae^{-i\frac{2\pi fx}{c}}+Be^{i\frac{2\pi fx}{c}}\\ \overset{\prime }{v}=\frac{1}{\rho c}\left(Ae^{-i\frac{2\pi fx}{c}}+Be^{i\frac{2\pi fx}{c}}\right) \end{array}$$

Since the energy conversion part of the fuze airflow-induced acoustic generator is at the bottom of the resonant cavity, only the longitudinal standing wave can make the piezoelectric transducer produce energy exchange, so only the longitudinal wave is considered. Mark the open end of the resonant cavity as the coordinate axis 0 point, then the bottom of the resonant cavity is *L*. Let the sound pressure of x=0 be $p_{1}$, the velocity of the particle is $v_{1}$, the sound pressure obtained at x=L is $p_{2}$, and the velocity of the particle is $v_{2}$, the natural frequency of the acoustic mode of the resonant cavity can be obtained by the formula of sound wave transmission.

(5) $$\left[\begin{array}{c} p_{2}\\ v_{2} \end{array}\right]=\left[\begin{array}{c} \cos kL\\ \frac{1}{\rho c}\sin kL \end{array}\begin{array}{c} -\rho c\sin kL\\ \cos kL \end{array}\right]\left[\begin{array}{c} p_{1}\\ v_{1} \end{array}\right]$$

According to the open end condition, the condition $p_{2}=0$ and the closed end condition $v_{2}=0$ are substituted, and the acoustic mode expression in the resonant cavity can be obtained as:

(6) $$f=\frac{(2k-1)c}{4L}\;\left(k=1,2,3\cdots \right)$$

where *f* is the acoustic mode frequency of a short resonant cavity with one end open and one end closed, and *k* represents the order of the acoustic mode.

According to the frequency correction study of the short resonant cavity, it is found that the diameter of the resonant cavity also has an effect on the size of the resonant frequency [26]. The frequency correction formula is obtained through experimental analysis, which can be expressed as:

(7) $$f=\frac{(2k-1)c}{4(L+\Delta L)}\;\left(k=1,2,3\cdots \right)$$

where ΔL=0.61R. 

Edge tone refers to the sharp pure sound that occurs when the high-speed fluid from a narrow slit flows to a sharp solid. Studies have found that the frequency of edge sound is directly proportional to the flow velocity of the fluid, and is related to the distance from the jet exit to the sharp object [27].

(8) $$f_{s}=\frac{v}{4X(1+M_{a})}$$

where $f_{s}$. represents the frequency of edge sound, *v* represents the velocity of the jet, *X* represents the distance from the nozzle to the wedge, and $M_{a}=\frac{v}{c}$ is the Mach number.

According to the working principle of the piezoelectric transducer [28], if the transducer’s strength is sufficient, regardless of the damage of the piezoelectric sheet, to generate more energy, it is necessary to be able to form an exciting force with large amplitude a high vibration frequency. Therefore, the transducer must reach the resonance state, that is, $f_{s}=f$, which can be obtained:

(9) $$\frac{v}{4X(1+M_{a})}=\frac{(2k-1)c}{4(L+\Delta L+\alpha X)}$$

Therefore, the conditions when the generator reaches the maximum output power are:

(10) $$\frac{X}{L+\Delta L}=\frac{v}{(2k-1)\left(c+v\right)}$$

That is, when the length of the resonance cavity (*L*), the confronting distance between the nozzle and the wedge (*X*), and the diameter of the resonance cavity (*R*), and the flow velocity (*V*) in the generator structural parameters satisfy Formula (10), a stable sound wave can be formed. 

## 4. Experimental Research on Acoustic Excitation Performance

In order to further the engineering design of the fuze airflow-induced acoustic generator, the orthogonal experiment design method is used to study the influence of parameters of the excitation mechanism under multiple factors and multiple levels. According to the four design parameters of the generator, an experimental plan is designed to analyze the amplitude and frequency of the sound wave generated by the excitation mechanism of the air-induced acoustic generator, and determine the sensitive parameter that generates the maximum electric energy. The change in the diameter of the resonant cavity will cause the structural size of the entire experimental system to change. Therefore, this article first studies the three factors of the annulus gap size *H*, the distance *X* from the resonant cavity to the annulus, and the length *L* of the resonant cavity.

### 4.1. Experimental Test System

**Figure 4:**
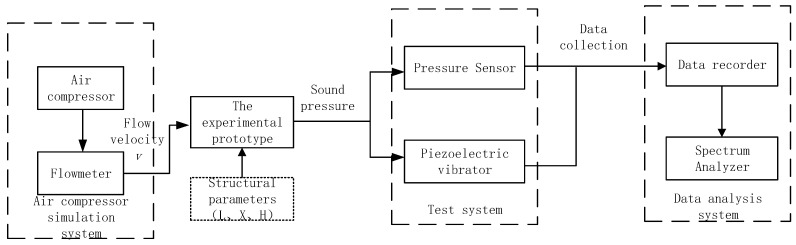
Block diagram of air compressor experimental test system.

The experimental test system is mainly composed of an air compressor simulation system, a test system and a data analysis system. The block diagram of the experimental test system is shown in Figure 4.

**Figure 5:**
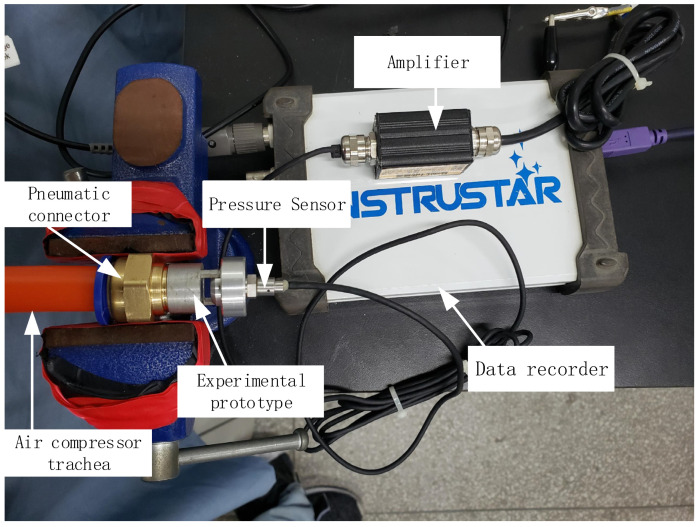
Test instrument.

Figure 5 is a photo of the test instrument. During the test, only the speed environment of the pipe flow is simulated. The air compressor has its own drying system, the flow meter can control the pipe flow speed, and the data recorder can record the voltage changes of the pressure sensor and the piezoelectric vibrator. During the test, the influence of factors such as temperature and humidity in the environment was ignored.

### 4.2. Orthogonal Factors and Experimental Design

According to the size of the fuze airflow-induced acoustic generator and the design requirements of the orthogonal test table, this paper carries out three levels of design for each factor. The annulus *H* is 0.5 mm, 1 mm, 1.5 mm, the resonant cavity length *L* is 8 mm, 9 mm, 10 mm, and the confronting distance *X* from the resonant cavity to the annulus is 2 mm, 3 mm, 4 mm, as shown in Table 1.

The experiment selects a 3-level 4-factor orthogonal table, namely $L_{9}\left(3^{4}\right)$. A total of nine experiments are required to analyze four factors including systematic errors, and three horizontal experiments are arranged for each factor, in which the fourth column is the error column, representing the influence caused by the systematic error. According to the principle of balanced collocation, three experiments are required for each level of each factor to ensure even level collocation. The orthogonal table design is shown in Table 2.

It can be seen from Table 2 that there are three experiments for each level of factor *A* (annular gap) among the nine experiments. The three levels of factor *A* (annular gap), factor*B*(resonant cavity length) and *C* (distance from resonant cavity to annulus) all appear once, so that when the experimental results resulting from the same level of factor *A* (annulus) are summed or averaged, the effects of the other factors are fixed. That is to say, the difference between the experimental results is due to the difference of the *A* factor (annular gap), which makes the levels of the *A* factor (annular gap) comparable. Similarly, the factor *B* (length of the resonant cavity) or factor *C* (the distance between the resonant cavity and the annulus) has similar properties, so the designed test table is reasonable.

### 4.3. Acoustic Signal Test and Test Eesult Analysis

When the fuze airflow-induced acoustic generator achieves the maximum output power, the edge sound must be consistent with the acoustic mode frequency of the resonant cavity.

According to Equation (10), the parameters in Table 2 are substituted in. The experiment is only carried out in the first-order mode, so *k* = 1, and the sound velocity *c* = 340 m/s is taken to obtain the flow velocity at the maximum output power of the corresponding nine groups of test prototypes, as shown in Table 3. Substitute the structural parameters in Table 2 into Equation (10), because the experiment is only carried out in the first-order mode, so *k* = 1. Taking sound velocity *c* = 340 m/s, the flow velocity corresponding to the maximum power output of nine groups of test prototypes can be obtained, as shown in Table 3.

**Figure 6:**
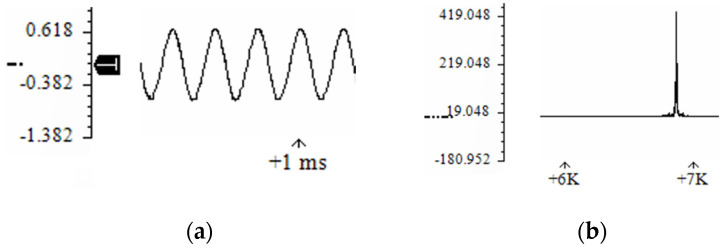
Sound pressure curve of fuze airflow-induced acoustic generator. (**a**) Sound pressure amplitude; (**b**) Sound pressure spectrum.

The air compressor blowing simulation experiment was carried out according to the calculated flow rate in Table 3. The sound pressure data of each group was amplified by the pulsating pressure sensor and recorded in the data recorder. The readout sound pressure data is shown in Figure 6.

The model of the pulsating pressure sensor in the experiment is CYY28, and the conversion relationship between the output pressure and the voltage signal is: x=0.05ΔP, where *x* (V) is the converted voltage, *P* (kPa) is the cavity bottom pressure. According to the sound pressure data measured in the experiment, after converting the voltage signals of the nine groups of test results into pressure, the orthogonal test results are shown in Table 4.

In Table 4, *K_ij_* represents the sum of the sound pressure amplitudes corresponding to the *i*-th level experiment of the *j*-th factor. *J_ij_* represents the sum of sound pressure frequencies corresponding to the *i*-th level experiment of the *j*-th factor. In the same column, *R* represents the range of sound pressure amplitude, and *Z* represents the range of sound pressure frequency.

(11) $$\{\begin{array}{c} k_{ij}=\frac{K_{ij}}{n}\\ j_{ij}=\frac{J_{ij}}{n} \end{array}$$

*k_ij_* represents the average value of the corresponding sound pressure amplitude in the experiment where the *i*-th level of the *j*-th factor is located. *j_ij_* represents the average value of the corresponding sound pressure frequency in the experiment where the *i*-th level of the *j*-th factor is located. *n* is the number of trials for each level of the factor, where *n* = 3.

$R_{j}$ represents the extreme difference of the sound pressure amplitude, $Z_{j}$ represents the extreme difference of the sound pressure frequency, the larger the range, the more obvious the influence of this factor on the test results.

(12) $$\{\begin{array}{c} R_{j}=\max\left\{k_{1j},k_{2j},k_{3j}\right\}-\min\left\{k_{1j},k_{2j},k_{3j}\right\}\\ Z_{j}=\max\left\{j_{1j},j_{2j},j_{3j}\right\}-\min\left\{j_{1j},j_{2j},j_{3j}\right\} \end{array}$$

According to the results in Table 4, the system error range of the sound pressure amplitude is: $R_{e}=17.82$, and the system error of the sound pressure frequency is: $Z_{e}=0.23$. The analysis results are as follows:

- Range order of sound pressure amplitude: $R_{A}=41.20>R_{C}=30.35>R_{B}=23.63$. They are all significantly greater than $R_{e}=17.82$. It shows that the three factors of *A* (annular gap size), *B* (resonant cavity length) and *C* (the distance between the nozzle and the resonant cavity) all have an impact on the sound pressure amplitude. According to the magnitude of the range, the primary and secondary order of the impact on the sound pressure amplitude is A, C, B. Therefore, the optimal combination for the sound pressure amplitude index is $A_{3}B_{1}C_{3}$.
- Range order of sound pressure frequency: $Z_{B}=3.83>Z_{A}=0.63>Z_{C}=0.03$. Only $Z_{A}$ and $Z_{B}$, greater than $Z_{e}=0.23$. It shows that *A* (annular gap size) and *B* (cavity length) have an influence on the frequency of sound pressure, while *C* (the distance between the nozzle and the resonant cavity) has less influence on the frequency of sound pressure than the systematic error. Therefore, it can be considered that *C* (the distance between the nozzle and the resonant cavity) has no influence on the frequency of sound pressure. According to the magnitude of the range, the order of the primary and secondary effects on the sound pressure frequency is *B*, *A*. Therefore, the optimal combination for the sound pressure amplitude index is $A_{3}B_{1}$.

The best combination of the two test indicators of comprehensive sound pressure amplitude and sound pressure frequency is: $A_{3}B_{1}C_{3}$.

## 5. Correction of Sound Pressure Frequency Empirical Formula

Let the amplitude main effects of the three levels of *A* factor be $\alpha_{1},{\;\alpha }_{2}$, and $\alpha_{3}$, and the frequency main effects are $o_{1}$, $o_{2}$, $o_{3}$. The main effects of the horizontal amplitude of *B* and *C* are $\beta_{1}$, $\beta_{2}$, $\beta_{3}$ and $\gamma_{1}$, $\gamma_{2}$, $\gamma_{3}$, and the main effects of the horizontal frequency of *B* and *C* are $m_{1}$, $m_{2}$, $m_{3}$ and $n_{1}$, n, $n_{3}$. $x_{ijk}$ represents the sound pressure amplitude under the horizontal combination $A_{i}B_{j}C_{k}$. $f_{ijk}$ represents the sound pressure frequency under the horizontal combination $A_{i}B_{j}C_{k}$. $\delta_{ijk}$ represents the random error of the sound pressure amplitude, and $\varepsilon_{ijk}$ represents the random error of the sound pressure frequency, so the statistical model can be expressed as:

(13) $$\{\begin{array}{c} \begin{array}{c} x_{ijk}=\mu +\alpha_{i}+\beta_{j}+\gamma_{k}\\ f_{ijk}=\theta +o_{i}+m_{j}+n_{k} \end{array}\\ \begin{array}{c} \alpha_{1}+\alpha_{2}+\alpha_{3}=\beta_{1}+\beta_{2}+\beta_{3}=\gamma_{1}+\gamma_{2}+\gamma_{3}\\ o_{1}+o_{2}+o_{3}=m_{1}+m_{2}+m_{3}=n_{1}+n_{2}+n_{3} \end{array} \end{array}$$

Then, the sum of squared deviations can be expressed as:

(14) $$\{\begin{array}{c} {SS}_{Tx}=\sum_{i=1}^{n}{\left(x_{i}-\bar{x}\right)}^{2}=\sum_{i=1}^{9}{\left(x_{i}-\bar{x}\right)}^{2}\\ {SS}_{Tf}=\sum_{i=1}^{n}{\left(f_{i}-\bar{f}\right)}^{2}=\sum_{i=1}^{9}{\left(f_{i}-\bar{f}\right)}^{2} \end{array}$$

where $SS_{Tx}$ represents the sum of total deviation squares of sound pressure amplitude, $SS_{Tf}$ represents the sum of total deviation squares of sound pressure frequency, $\bar{x}$ represents the mean value of sound pressure amplitude, $\bar{f}$ represents the mean value of sound pressure frequency, $x_{i}$ represents the sound pressure amplitude of the *i*-th group of experiments, and $f_{i}$ represents the sound pressure frequency of the *i*-th group of experiments.

The sum of squared deviations of the amplitude and frequency of factor *A* can be expressed as:

(15) $$\{\begin{array}{c} S_{Ax}=\sum_{i=1}^{n}m_{i}{\left(x_{i}-\bar{x}\right)}^{2}=3\sum_{i=1}^{3}{\left(x_{i}-\bar{x}\right)}^{2}\\ S_{Af}=\sum_{i=1}^{n}m_{i}{\left(f_{i}-\bar{f}\right)}^{2}=3\sum_{i=1}^{3}{\left(f_{i}-\bar{f}\right)}^{2} \end{array}$$

In the formula, $m_{i}$ represents the number of experiments performed by factor *A* at the *i*-th level.

Substituting the calculation results in Table 4 to obtain:

$$\{\begin{array}{c} \begin{array}{c} \bar{x}=26.1\\ \bar{f}=6.33 \end{array}\\ \begin{array}{c} {SS}_{Tx}=5379.15\\ {SS}_{Tf}=2.735 \end{array}\\ \begin{array}{c} S_{Ax}=2583.03\\ S_{Af}=0.62 \end{array} \end{array}$$

The same method is used to calculate the sum of squared deviations of factors *B*, *C* and systematic errors. The data obtained are shown in Table 5.

The contribution rate of each factor to the sound pressure amplitude and frequency and the *F* ratio are further calculated according to the sum of square deviations of each factor. When the *F* value is greater than 1, it indicates that the change of the factor level has an impact on the experimental indicators. The calculation results are shown in Table 6.

According to the results in Table 6, the system error contribution rate of the sound pressure amplitude is 8.86%, and the system error of the sound pressure frequency is 3%. The analysis results are as follows:

- The order of the contribution rate of the sound pressure amplitude: *A* > *C* > *B* > systematic error (blank column). Therefore, the three parameters all have an impact on the sound pressure amplitude. Among them, the annulus has the greatest effect on the sound pressure amplitude, followed by the confronting distance and the length of the resonant cavity. So the optimal combination of sound pressure amplitude is: $A_{3}B_{1}C_{3}$.
- The order of the contribution rate of the sound pressure frequency: *B* (resonant cavity length L) > *A* > System error (Error column) > C. Therefore, the length of the resonant cavity is the most sensitive parameter that affects the sound pressure frequency, followed by the annular gap. Because the effect of the confronting distance on the frequency is less than the system error, it can be considered that the spacing has no effect on the sound pressure frequency.

Therefore, the optimal combination of sound pressure frequency is: $A_{3}B_{1}$.

In summary, the optimal combination of the excitation mechanism of the fuze airflow-induced acoustic generator is: $A_{3}B_{1}C_{3}$. The experimental results are the same as those of range analysis.

According to the experimental results in Table 6, Formula (7) is modified to introduce the influence of the size of the annulus on the acoustic resonance frequency. According to the contribution rate of each parameter to the influence of the sound pressure frequency, we can obtain:

$$\frac{\mathrm{Contribution}\;\mathrm{rate}\;\mathrm{of}\;\mathrm{annulus}\;\mathrm{to}\;\mathrm{the}\;\mathrm{sound}\;\mathrm{pressure}\;\mathrm{frequency}}{\mathrm{Contribution}\;\mathrm{rate}\;\mathrm{of}\;\mathrm{resonant}\;\mathrm{cavity}\;\mathrm{to}\;\mathrm{sound}\;\mathrm{pressure}\;\mathrm{frequency}}=\frac{22.63\%}{74.31\%}=0.3$$

Therefore, it can be assumed that the influence factor of the annulus on the sound pressure frequency is *α* = 0.3, and the correction formula is proposed as follows:

(16) $$f=\frac{(2k-1)c}{4(L+\Delta L+\alpha H)}$$

This paper only studies the frequency of the first-order acoustic mode, so substituting *k* = 1 into Formula (17), we can get:

(17) $$f=\frac{c}{4(L+\Delta L+\alpha H)}$$

For the nine groups of experiments in Table 2, compare the theoretical and experimental values of the frequencies of Formula (6), Formula (7), and Formula (17) to verify the validity of the frequency Formula (17) proposed in this paper. In Table 7, *T* represents the theoretical sound pressure frequency, *A* represents the actual sound pressure frequency, and *D* represents the relative error.

According to Table 7, it can be found that the maximum relative error of Formula (6) is as high as 62.21%, the maximum relative error of Formula (7) is 18.86%, and the maximum relative error of Formula (17) is 13.69%. Therefore, the revised Formula (17) can improve the estimation accuracy of the resonance frequency of the airflow-induced acoustic generator.

## 6. Conclusions

In this paper, through experiments, the sensitive parameters of the dynamic excitation of the airflow-induced acoustic generator are determined. The results show that the size of the annulus is the most sensitive parameter that affects the sound pressure amplitude, followed by the confronting distance and the length of the resonant cavity. However, the length of the resonant cavity is the most sensitive parameter that affects the sound pressure frequency, the size of the annulus is more sensitive to the sound pressure frequency, and the spacing has almost no effect on the sound pressure frequency.

This paper also proposes a correction formula for estimating the vibration frequency of the airflow-induced acoustic generator, adding the influence of the size of the annulus on the vibration frequency, and the influence factor *α* = 0.3 is determined through experiments. The corrected frequency estimation empirical formula has the smallest error between the theoretical value and the experimental value. It can be used as an effective method for estimating the vibration frequency and a reference basis for the design of the airflow acoustic generator and provide a design scheme for a universal fuze power supply.

## Figures and Tables

**Table 1:** Structural parameters of fuze airflow-induced acoustic generator.

	Factor	H (mm) (A)	L (mm) (B)	X (mm) (C)
Level				
1		0.5	8	2
2		1	9	3
3		1.5	10	4

**Table 2:** Orthogonal table of mechanical parameters of fuze airflow-induced acoustic generator.

		Factor	A	B	C	Error Column
	Level					
Experiment Number						
1			1	1	1	1
2			1	2	2	2
3			1	3	3	3
4			2	1	2	3
5			2	2	3	1
6			2	3	1	2
7			3	1	3	2
8			3	2	1	3
9			3	3	2	1

**Table 3:** Test conditions at maximum power.

Level	*A*_1_ *B*_1_ *C*_1_	*A*_1_ *B*_2_ *C*_2_	*A*_1_ *B*_3_ *C*_3_	*A*_2_ *B*_1_ *C*_2_	*A*_2_ *B*_2_ *C*_3_	*A*_2_ *B*_3_ *C*_1_	*A*_3_ *B*_1_ *C*_3_	*A*_3_ *B*_2_ *C*_1_	*A*_3_ *B*_3_ *C*_2_
Velocity (m/s)	75.14	112.71	150.28	126.71	168.94	61.54	192.91	67.66	101.49

**Table 4:** Orthogonal experiment results.

		Factor	A 1	B 2	C 3	Error Column 4	Experimental Result	
	Level						Amplitude *x* (kPa)	Frequency *f* (kHz)
Experiment Number								
1			1	1	1	1	5.66	6.55
2			1	2	2	2	6.62	6.11
3			1	3	3	3	8.5	5.48
4			2	1	2	3	28.74	6.87
5			2	2	3	1	28.68	6.06
6			2	3	1	2	12.28	5.83
7			3	1	3	2	86.42	7.38
8			3	2	1	3	14.62	6.64
9			3	3	2	1	43.34	6.01
Amplitude		*K*_1*j*_	20.78	120.82	32.56	77.68	$\sum x_{i}=234.86$	
		*K*_2*j*_	69.7	49.92	78.7	105.32		
		*K*_3*j*_	144.38	64.12	123.6	51.86		
		*k*_1*j*_	6.93	40.27	10.85	25.89		
		*k*_2*j*_	23.23	16.64	26.23	35.11		
		*k*_3*j*_	48.13	21.37	41.2	17.29		
		$R_{j}$	41.20	23.63	30.35	17.82		
Frequency		*J*_1*j*_	18.14	20.8	19.02	18.62		$\sum f_{i}=56.93$
		*J*_2*j*_	18.76	18.81	18.99	19.32		
		*J*_3*j*_	20.03	17.32	18.92	18.99		
		*j*_1*j*_	6.05	6.93	6.34	6.21		
		*j*_2*j*_	6.25	6.27	6.33	6.44		
		*j*_3*j*_	6.68	5.77	6.31	6.33		
		$Z_{j}$	0.63	3.83	0.03	0.23		

**Table 5:** The sum of squared deviations of each factor.

	Factor	A		B		C		Error Column	
Result		*x* (kPa)	*f* (kHz)	*x* (kPa)	*f* (kHz)	*x* (kPa)	*f* (kHz)	*x* (kPa)	*f* (kHz)
$y_{1}$		20.78	18.14	120.82	20.8	32.56	19.02	77.68	18.62
$y_{2}$		69.7	18.76	49.92	18.81	78.7	18.99	105.32	19.32
$y_{3}$		144.38	20.03	64.12	17.32	123.6	18.92	51.86	18.99
s		2583.03	0.62	938.15	2.03	1381.47	0.02	476.51	0.08

**Table 6:** Analysis of variance.

Factor	Deviation Sum of Squares		Degree of Freedom		F		Rate of Contribution	
	*x* (kPa)	*f* (kHz)	*x* (kPa)	*f* (kHz)	*x* (kPa)	*f* (kHz)	*x* (kPa)	*f* (kHz)
A	2583.03	0.619	2	2	5.42	7.55	48.02%	22.63%
B	938.15	2.032	2	2	1.97	24.67	17.44%	74.31%
C	1381.47	0.002	2	2	2.9	0.02	25.68%	0.06%
Error column	476.51	0.082	2	2			8.86%	3.00%
Sum	5379.15	2.735	8	8				

**Table 7:** Theoretical and experimental values of frequency empirical formula.

	H (mm)	L (mm)	X (mm)	A (kHz)	Formula (6)		Formula (7)		Formula (17)	
					T (kHz)	D (%)	T (kHz)	D (%)	T (kHz)	D (%)
1	0.5	8	2	6.55	10.63	62.21	7.17	17.44	7.08	13.69
2	0.5	9	3	6.11	9.44	54.57	6.42	15.45	6.34	12.30
3	0.5	10	4	5.48	8.50	55.11	5.80	18.86	5.74	14.90
4	1	8	3	6.87	10.63	54.66	6.94	11.97	6.77	8.27
5	1	9	4	6.06	9.44	55.85	6.23	16.40	6.09	11.95
6	1	10	2	5.83	8.50	45.80	6.14	11.72	6.01	8.43
7	1.5	8	4	7.38	10.63	43.97	6.72	4.23	6.49	0.15
8	1.5	9	2	6.64	9.44	42.24	6.61	6.23	6.39	2.35
9	1.5	10	3	6.01	8.50	41.43	5.96	8.38	5.78	4.55
